# Stress reactions after a patient suicide and their relations to the profile of mental health professionals

**DOI:** 10.1186/s12888-015-0655-y

**Published:** 2015-10-28

**Authors:** Dolores Angela Castelli Dransart, Jean-Luc Heeb, Alida Gulfi, Elisabeth M. Gutjahr

**Affiliations:** HES-SO: University of Applied Sciences and Arts Western Switzerland, School of Social Work Fribourg (HETS-FR), Rue Jean Prouvé 10, 1762 Givisiez, Switzerland

**Keywords:** Suicide, Patient suicide, Stress reactions after patient suicide, IES-R, Profiles of professionals, Support, Training

## Abstract

**Background:**

Patient suicide is a professional hazard for mental health professionals and an event likely to trigger stress reactions among them.

This study aimed to identify typical profiles of professionals after a patient suicide to address the severity of stress reactions and its discriminant variables.

**Methods:**

Mental health professionals (*N* = 666) working in institutional settings or private practice in the French-speaking part of Switzerland filled out a self-administered questionnaire including the IES-R (Impact of Event Scale-Revised). Profiles were identified by cluster analysis.

**Results:**

The interplay of variables pertaining to the relationship to the patient, exposure to suicide, support and training contributed to explaining the severity of stress reactions after a patient suicide. Five profiles of professionals were identified. Low-impacted professionals (55.8 % of the sample) were characterised either by high support and anticipation (*anticipators with support*), emotional distance to the patient (*distant professionals*) or no contact with the patient at the time of death (*no more contact with patient professionals*). Emotional closeness to, and responsibility for the patient were typical of moderately-impacted professionals (36.6 %, *concerned professionals*), while highly-impacted professionals felt emotionally close to the patient and lacked support although more than half of them sought it (7.7 %, *unsupported professionals*).

**Conclusions:**

Differences in the professionals’ profiles relate prominently to the interplay between risk and protective factors. Professionals who were appropriately supported, i.e., according to their risk profile, were able to cope with the event. Taking into account the profiles of professionals and the severity of stress reactions may enable the screening of those professionals most in need of support. Those most impacted sought out help more frequently. However, only a minority of them were offered sufficient support. Institutional or vocational bodies should take measures to ensure that professionals seeking help find it easily and promptly.

The combination of training and support seems to be crucial for mitigating risk factors since the three low impacted subgroups had received the most training and support.

## Background

Patient suicide is a critical and stressful event [1]. Since the first landmark studies in the 1980s [2, 3] it has been considered as a professional hazard for caregivers working with patients suffering from mental health disorders or experiencing other psycho-social or socio-economic difficulties. Stress reactions or traumatic symptoms following a patient suicide have been examined in a number (14) of studies between 1988 and 2014 using the Impact of Event Scale [4] or the Impact of Event Scale-Revised [5]. Findings on the severity of stress reactions are heterogeneous: some studies found that a minority (7–14 %) of respondents reported symptoms above the cut off scores used in the literature (>19 for IES and ≥ 25 for IES-R [1, 6, 7]), while others found markedly higher percentages (49–53 % [2, 3, 8]).

Some studies have investigated predictors of severity for stress reactions, however findings pertaining to patients or professionals' characteristics have been inconsistent [6]. The length and intensity of the relationship with the patient proved to be significant, i.e., professionals who felt closer to, or were in a therapeutic relationship with the patient for longer reported higher levels of stress [6, 9, 10]. Support was identified as a protective factor [10]. Results concerning vocations were inconsistent among studies using the IES and the IES-R [6, 9, 10]. To our knowledge, no study so far has purposely investigated subgroups of professionals likely to be more vulnerable to stress reactions by taking into account the potential interaction of various variables. Hence this paper aims (i) to check whether typical profiles can be identified to classify professionals into distinct subgroups when considering the predictors of stress reactions evidenced in the literature, (ii) to examine how these typical profiles relate to the severity of stress reactions after a patient suicide; (iii) to evidence the variables that most discriminate the typical profiles in order to identify prevention and postvention measures.

## Methods

### Data

Cross-sectional data were collected anonymously by means of standardised self-administered paper-pencil questionnaires in 2006 and 2009.

Pooled data sources from two studies on personal and professional consequences of patient suicide among mental health and social professionals in French-speaking Switzerland were used. The first source comprised data from professionals working in institutional settings such as psychiatric hospitals, outpatient psychiatric services, social and medical services, residential homes for persons with mental health or addiction disorders, homes for the elderly and prisons. Data were collected among psychiatrists, nurses, nursing auxiliaries, psychologists, social educators and social workers in the cantons of Fribourg, Geneva and Vaud. The second source investigated psychiatrists and psychologists working in private practice in the cantons of Fribourg, Geneva, Jura, Neuchâtel, Valais, and Vaud.

Data for professionals in institutional settings were obtained using a two-stage survey. First, 767 institutions were identified in public directories and sent a short written questionnaire to assess whether they had experienced a patient suicide in the 5 years prior to the survey. Of the 521 (67.9 %) institutions that answered the questionnaire, 152 (29.2 %) had experienced a patient suicide and a majority (92.1 %, *n* = 140) of them agreed to participate in the second stage of the study. A total of 5123 self-administered written questionnaires were then sent to these institutions to be filled out by professionals. In complex organisational settings, local referents were trained to encourage participants' involvement, thereby improving the response rate. Out of the 1211 (23.6 %) professionals who sent back the questionnaire, 704 (58.1 %) reported having experienced a patient suicide. Respondents with lacking data for central study variables (IES-R, cluster variables) were excluded and a final sample of 558 professionals in institutional settings was obtained.

A one-stage survey was used to collect data on therapists in private practice. All members of professional societies (except for the society of psychiatry in Valais, which refused to participate) in the investigated cantons at the time of the study, i.e., 769 psychiatrists and 765 psychologists, received the main self-administrated written questionnaire with the same core variables as for professionals in institutional settings. Of the 415 (27.0 %) therapists who returned the questionnaire, 158 (38.1 %) had been confronted with a patient suicide. After excluding professionals with missing data, the final sample comprised 108 therapists working in private practice.

Pooling the data sources yielded a final sample of 666 mental health and social caregivers. Participation was voluntary, in accordance with the request from the Ethics Committee of the University Hospitals of Lausanne and Geneva, which approved the studies. Consent to the survey was assumed for those completing and returning the questionnaire.

### Measures

The self-administered questionnaire included 60 questions and 9 scales adapted from a previous Canadian study [11]. The variables used in this article were addressed for professionals working in both institutional settings and private practice.

Stress reactions after a patient suicide were assessed using the validated French version of the Impact of Event Scale-Revised [12]. The IES-R is not to be considered as a proxy for diagnosis or be used as a diagnostic instrument, especially when integrated in a self-administered questionnaire. The IES-R comprises 22 items measuring symptoms of intrusion (8 items including intrusive thoughts, nightmares, intrusive feelings and imagery, dissociative-like re-experiencing), avoidance (8 items measuring numbing of responsiveness, avoidance of feelings, situations and ideas), and hyperarousal (6 items measuring anger, irritability, hypervigilance, difficulty concentrating, heightened startle response). Participants were asked to indicate on a 5-point scale, ranging from *not at all* (0) to *extremely* (4), how distressing each item had been for them during the month following the patient suicide. Subscale scores for intrusion (ranging from 0 to 32), avoidance (ranging from 0 to 32) and hyperarousal (ranging from 0 to 24) were obtained by adding up the corresponding item scores. The subscale scores added up to the IES-R's total score. The cut off score of 25 found in the literature [1] was chosen. However, this value does not correspond to any clinical diagnosis. The adoption of a cut off was not intended for clinical purposes but as a way of characterising and comparing subgroups for the cluster analysis.

Respondents faced with more than one patient suicide were asked to consider the most recent one. The psychometric properties of the IES-R were found to be satisfactory in a subsample of the data used in this study [13].

Based on the literature [14] on the predictors of a patient suicide's impact, variables were selected to determine professionals’ profile in terms of relationship with the patient (variables of last contact with patient, responsibility for the patient, still in contact with him/her at the time of suicide, length of relationship, closeness to the patient), exposure to suicide (previous suicide attempts, suicide expected, having seen the body at the suicide scene), support (support sought, support received), and training (theoretical training and clinical training). Additionally, socio-demographic characteristics of the respondents (gender, age, profession, work setting, years of professional experience) were considered to provide a more accurate description of the professional’s profile.

### Statistical analysis

Data were analysed using SPSS version 19.0 [15]. Statistical procedures included sample description, classification of respondents into subgroups and characterisation of the evidenced subgroups. As a classification technique, cluster analysis aims at partitioning a sample into mutually contrasted and internally homogenous subgroups, i.e., whether and how the respondents can be classified into a few distinct groups according to similar professional’s profiles. A combination of hierarchical and non-hierarchical techniques was used [16]. Firstly, hierarchical clustering (Euclidian distance, Ward’s method) was carried out to get an initial partition. The number of subgroups retained was based on both statistical or visual (variance explained, dendrogram) and practical criteria (subgroup size). Secondly, the initial partition evidenced by the hierarchical analysis was refined by non-hierarchical clustering (k-means). The stability of the classification was tested by varying the data order and by cross-validation (comparison of the separate clustering of the randomly split sample). Variables included in the cluster analysis were related to the relationship with the patient, exposure to suicide, support, training, and socio-demographic characteristics (see above). To characterise the stress reactions of the professional’s profiles, subgroup membership was considered as a predictor and the IES-R scores as a criterion. Analysis of variance was further used to examine the effects of subgroup membership on the IES-R scores. Further differences between subgroups were addressed by analysis of variance (continuous variables) and chi-square tests (dichotomous variables).

To date, research on the variations of stress reactions following a patient suicide was mainly of a descriptive nature or based on regression analysis. Descriptive results aim to address levels of stress reactions by subgroups according to factors such as gender, age or relationship with the patient within a given sample, while studies based on regression techniques measure the individual contribution of different predictors to the variation of the stress reactions when they are analysed together. Both approaches rely on a factorial design. Due to limited sample sizes, factorial designs usually fail to provide insight into profiles of individuals, i.e., how their characteristics are related. Cluster analysis, however, overcomes this shortcoming.

## Results

### Participants and patients’ characteristics

64.7 % of the 666 professionals were women and 98.8 % had completed their education. Mean age at the time of the investigation was 45.7 years (SD = 10.2), and the average length of professional practice was 18.9 years (SD = 10.1). 83.8 % of professionals worked in institutions: 39.6 % were nurses, 26.2 % psychiatrists, 14.5 % social workers, 9.7 % psychologists, 5.1 % social educators, 2.3 % nurses auxiliaries, and 2.6 % other professionals.

Most respondents had faced more than one patient suicide during their career (M = 2.7, SD = 1.4). The mean time since the last patient suicide was 4.2 years (SD = 5.3). For 49.8 % of participants, the patient was under their responsibility. 60.2 % of participants were still in contact with the patient at the time of death and the mean length of relationship with him/her was 1.7 years (SD = 2.7). 4.2 % last saw the patient at the time of suicide, 57.3 % met him/her up to one week prior to the suicide, 20.7 % from one week to a month prior to the suicide. 51.8 % reported feeling moderately to strongly close to the deceased patient. 42.3 % of professionals declared having received theoretical education and 26.7 % clinical training in suicide prevention. 39.2 % of respondents had actively sought psychological and/or social support after the patient suicide and 74.3 % reported having received sufficient support.

Participants felt well at the time of the study (M = 2.1, SD = 0.8; ranging from 1 *very well* to 5 *very bad*) and not overstressed (M = 2.2, SD = 0.7; ranging from 1 *low* to 4 *excessive*). They were satisfied with their job (M = 7.8, SD = 1.5; ranging from 1 *not at all satisfied* to 10 *very satisfied*) and with their relationship with colleagues (M = 3.5, SD = 0.6, ranging from 1 *unsatisfied* to 4 *satisfied*). No significant differences were observed between participants who had been faced with a patient suicide and their colleagues who had not.

Concerning the deceased patient, 53.7 % were men. Among all patients, 79.9 % had a mental health disorder, 28.3 % had an addiction disorder, and 35.2 % had psycho-social and socio-economic difficulties at the time of suicide (multiple responses possible). Of all patients, 22.8 % completed suicide within the institutional setting and 46.5 % had already made a suicide attempt. 54.2 % of professionals knew about suicidal ideation at time of suicide and 17.4 % discovered the deceased’s body.

### Identifying subgroups and stress reactions

Hierarchical cluster analysis suggested five subgroups on the basis of the variables pertaining to the professionals’ profiles. This classification was the most suitable, as a lower number of subgroups resulted in the aggregation of distinct subgroups while further partitioning of the clusters produced numerous small subgroups that differed only slightly. After refinement of the initial classification by non-hierarchical cluster analysis, 8.6 % of the respondents changed subgroup.

Variations in the total score of the IES-R, used as a criterion, were closely related to subgroup membership (Table 1). Three low impacted subgroups, one moderately impacted subgroup and one highly impacted subgroup were found. The three low impacted subgroups accounted for more than half of the sample (55.8 %) while 36.6 % were in the moderately impacted subgroup and the smallest subgroup (7.7 %) was the highly impacted one. Intrusion, avoidance and hyperarousal scores varied significantly across subgroups as did the total score on the IES-R. The share of professionals with an IES-R total score of 25 or higher varied strongly among subgroups, as one-third of the respondents in the highly impacted subgroup and less than 3.2 % in the lowest impacted subgroup were concerned. 14 % of the sample scored 25 or above on the IES-R.

**Table 1:** IES-R total and subscale mean scores by subgroups

Subgroup	Impact	Total IES-R mean score [SD]	Intrusion mean score [SD]	Avoidance mean score [SD]	Hyperarousal mean score [SD]	Total IES-R score ≥25	Size (n)	Size (%)
A	Low	8.6 [8.3]	5.2 [4.2]	2.3 [2.9]	1.0 [2.6]	3.2 %	63	9.5 %
B	Low	9.8 [9.6]	5.3 [4.6]	3.1 [4.2]	1.5 [2.8]	8.8 %	151	22.7 %
C	Low	11.1 [11.7]	6.0 [5.2]	3.7 [5.0]	1.4 [3.3]	9.7 %	157	23.6 %
D	Moderate	16.0 [11.9]	9.4 [6.0]	4.3 [4.5]	2.3 [3.1]	19.0 %	244	36.6 %
E	High	21.2 [13.4]	11.4 [6.4]	6.2 [5.0]	3.6 [4.4]	33.3 %	51	7.7 %
Total		13.1 [11.7]	7.4 [5.8]	3.8 [4.5]	1.9 [3.2]	14.0 %	666	100.0 %
Differences between subgroups (p)		<0.001^a^	<0.001^a^	<0.001^a^	<0.001^a^	<0.001^b^		

IES-R scores are the criterion, subgroup membership is the predictor

^a^one-way analysis of variance

^b^chi-square test

Percentages may not total 100 due to rounding

Subgroup variations in the total score of the IES-R are in part related to the predictors of stress reactions after a patient suicide and to the profession [6, 9, 10]. Mean scores of the IES-R were higher for emotional closeness to the patient (M = 15.8; no closeness M = 10.3), insufficient support received (M = 17.6; sufficient support M = 11.6) and nurses (M = 14.4) as well as educators (M = 14.1; psychiatrists M = 12.0, social workers M = 11.3, psychologists M = 10.7). The percentage of variance explained by subgroup (one-way analysis of variance of the total score, R^2^ = 9.6 %, *p* <0.001) and by closeness to the patient, support received, and profession (four-way analysis of variance, R^2^ = 12.7 %, *p* <0.001; nurses auxiliaries and other professionals combined due to small sample sizes) was in the same range. It increased slightly when considering all predictors simultaneously (five-way analysis of variance, R^2^ = 14.5 %, *p* <0.001) with significant contributions from all factors (subgroup η^2^ = 1.9 %, *p* = 0.010; support received η^2^ = 2.7 %, *p* <0.001; profession η^2^ = 2.7 %, *p* = 0.003) but closeness to the patient (η^2^ = 0.0 %, *p* = 0.596).

### Professionals’ profiles and subgroups

Differences in the professionals' profiles between the identified subgroups – significant for all characteristics but years of professional experience – relate prominently to the relationship with the patient and the support received (Tables 2, 3 and 4). Low-impacted professionals are characterised either by high support and anticipation of the suicide (*anticipators with support*, subgroup A), emotional distance to the patient (*distant professionals*, subgroup B) or no contact with the patient at the time of death (*no more contact with patient professionals*, subgroup C). Emotional closeness to, and responsibility for the patient are typical of moderately-impacted professionals (*concerned professionals*, subgroup D), while highly-impacted professionals lacked support (*unsupported professionals*, subgroup E).

**Table 2:** Professionals' profiles by subgroups: relationship with the patient

Subgroup	Last contact before suicide 24 h or less	Responsibility for patient	Still in contact with patient	Mean length of relationship (years)	Closeness to patient
A anticipators with support	17.5 %	23.8 %	12.7 %	1.8	31.7 %
B distant professionals	41.7 %	61.6 %	100.0 %	1.2	0.0 %
C no more contact with patient professionals	22.9 %	18.5 %	0.0 %	1.0	19.1 %
D concerned professionals	34.4 %	76.2 %	91.8 %	2.4	100.0 %
E unsupported professionals	31.4 %	17.6 %	35.3 %	1.2	100.0 %
Total	31.5 %	49.8 %	60.2 %	1.7	51.8 %
Differences between subgroups (p)	<0.001^a^	<0.001^a^	<0.001^a^	<0.001^b^	<0.001^a^

Percentages and mean indicate the value within each subgroup

^a^chi-square test

^b^one-way analysis of variance

**Table 3:** Professional’s profiles by subgroups: exposure to suicide, support and training

Subgroup	Exposure to suicide				Support		Training	
	Mean number of suicide during career	Patient’s previous suicide attempt	Suicide expected	Saw body at suicide scene	Support sought	Sufficient support received	Theoretical training	Clinical training
A anticipators with support	2.7	59.0 %	100.0 %	12.8 %	26.2 %	87.3 %	41.0 %	26.8 %
B distant professionals	2.4	41.2 %	13.2 %	23.0 %	38.4 %	76.8 %	49.0 %	27.6 %
C no more contact with patient professionals	2.6	42.4 %	0.0 %	14.4 %	23.7 %	76.4 %	45.2 %	30.2 %
D concerned professionals	2.7	49.2 %	25.4 %	17.3 %	50.0 %	83.6 %	38.6 %	25.9 %
E unsupported professionals	3.2	46.5 %	31.4 %	17.1 %	54.0 %	0.0 %	33.4 %	16.3 %
Total	2.7	46.5 %	24.2 %	17.4 %	39.2 %	74.3 %	42.3 %	26.7 %
Differences between subgroups (p)	0.021^a^	<0.001^b^	<0.001^b^	0.007^b^	<0.001^b^	<0.001^b^	0.010^b^	<0.009^b^

Percentages and mean indicate the value within each subgroup

^a^one-way analysis of variance

^b^chi-square test

**Table 4:** Professionals' profiles by subgroups: gender, age, profession, work setting, and experience

Subgroup						
	Men	Mean age (years)	Psychiatrists	Nurses	Work setting: institution	Mean length of professional experience experience (years)
A anticipators with support	31.7 %	43.1	17.5 %	52.4 %	92.1 %	17.7
B distant professionals	35.8 %	44.7	31.8 %	37.1 %	84.1 %	17.9
C no more contact with patient professionals	23.7 %	44.4	19.1 %	44.6 %	87.3 %	18.1
D concerned professionals	45.5 %	47.9	32.4 %	28.7 %	77.5 %	20.2
E unsupported professionals	25.5 %	46.0	11.8 %	64.7 %	92.2 %	19.7
Total	35.3 %	45.7	26.1 %	39.3 %	83.8 %	18.9
Differences between subgroups (p)	<0.001^a^	<0.001^b^	<0.001^a^	<0.001^a^	0.006^a^	0.120^b^

Percentages and means indicate the value within each subgroup

^a^chi-square test

^b^one-way analysis of variance

In the low-impact *anticipators with support* subgroup (A), the great majority of individuals received sufficient support after the patient suicide (87.3 %). More often than in other subgroups, the patient had already made a suicide attempt (59.0 %) and all professionals expected the suicide. On the contrary, the share of professionals who discovered or saw the deceased patient's body (12.8 %) was the lowest of all subgroups. Less than 1/3 of respondents felt close to the patient. 12.7 % were in contact with the patient at the time of death and 17.5 % in the 24 h preceding the suicide. Professionals working in an institution were overrepresented (92.1 %).

In the low-impact *distant professionals* subgroup (B), no respondent reported feeling close to the patient, although all of them were still in contact with him/her at the time of death (highest percentage) and 41.7 % had had the last contact in the 24 h preceding the suicide (highest percentage). Of all subgroups, these respondents saw the body at the suicide scene the most (23.0 %). Professionals in this subgroup received more theoretical education (49.0 %) than their colleagues in other subgroups; 27.6 % had clinical training in risk assessment of suicide. 31.8 % of professionals in this group were psychiatrists (second highest percentage).

In the low impact *no more contact with patient professionals* subgroup (C), all professionals had no more contact with the patient at the time of death and the length of the relationship to the patient was the shortest (M = 1.0 year). Almost one in five professional reported feeling close to the patient (19.1 %) or having been responsible for him/her in the past (18.5 %). Although professionals in this subgroup sought support less often (23.7 %) than those in the other subgroups, about ¾ (76.4 %) of them reported having received sufficient support. This subgroup had received clinical training more often than the other subgroups (30.2 %) and ranged at the second place for theoretical education (45.2 %). Women were overrepresented in this subgroup (76.3 %).

In the moderate impact *concerned professionals* subgroup (D) all professionals felt close to the patient and more than ¾ (76.2 %) were responsible for the patient (highest percentage of all groups). 9 professionals out of 10 (91.8 %) were still in contact with the patient at the time of death and they had, in average, the longest relationship with the patient of all subgroups (M = 2.4 years). Half of professionals sought support (50.0 %) after the patient suicide and 83.6 % reported having received sufficient support. Finally, 45.5 % were men (highest percentage), 32.4 % were psychiatrists (highest percentage), while 28.7 % were nurses (lowest percentage) and 77.5 % worked in an institutional setting (lowest percentage).

In the high impacted *unsupported professionals* subgroup (E) the support received was qualified as insufficient by all professionals, although 54.0 % of them sought such support. They all felt close to the deceased patient, but only a minority (17.6 %, lowest percentage) were responsible for her/him. Professionals in this subgroup faced more patient suicides than in other subgroups (3.2 %, highest percentage). They received less theoretical education (33.4 %) and clinical training (16.3 %) than their colleagues in other subgroups. This subgroup comprised the smallest percentage of psychiatrists (11.8 %), but the highest percentage of nurses (64.7 %) and professionals working in institutional settings (92.2 %).

## Discussion

This study investigates the profiles of mental health and social caregivers regarding patient-caregiver relationship, exposure to suicide, support, training and professional characteristics, and their link with the severity of stress reactions following a patient suicide. While research has previously focused on describing reactions and identifying single predictors through regression analysis, this study adopted a comprehensive view by exploring the interplay of predictors as they combine within distinct profiles by means of cluster analysis.

Five distinct subgroups of professionals both in terms of profiles - mainly emphasising patient-caregiver relationship and support - and stress reactions were found: *anticipators with support*, *distant professionals*, *no more contact with patient professionals, concerned professionals*, and *unsupported professionals*. The three subgroups, *anticipators with support*, *distant professionals* and *no more contact with patient professionals* accounted for more than half (55.8 %) of the sample and had low stress reactions, while the *concerned professionals* subgroup presented a moderate impact (36.6 %) and the *unsupported professionals* subgroup (7.7 %) a high impact. These findings might explain previous results reporting contrasting proportions of respondents with high scores on the IES (or the IES-R) [1–3, 6–8]. Indeed, presuming that these various subgroups are to be found in other studies and contexts, the proportion of professionals with higher scores on the IES (or the IES-R) could vary in accordance with the size of the various subgroups. For example, in contexts and settings were professionals would receive less support than our Swiss sample (where ¾ of professionals reported having received sufficient support) the *unsupported professionals* subgroup might be larger and have a greater impact on the mean IES (or IES-R) score of the whole sample.

Furthermore, our results shed new light on, and question previous knowledge with regard to risk and protective factors.

According to the literature, variables pertaining to the patient-caregiver relationship (such as emotional closeness, length of relationship, being responsible for patient) or to the exposure to suicide (such as number of suicides experienced, having seen the body) are considered as risk factors [6, 10, 14, 17], while support and training in suicide prevention and crisis intervention seem to serve as protective factors [6, 10, 18, 19]. Although our results confirm that these factors play an influential role when addressing stress reactions after a patient suicide, their combination in different profiles suggests that their effects are not additive. For instance, the percentage of professionals who felt close to the patient varies strongly across the three low impact subgroups. Though the *anticipators with support* showed the highest percentage, adequate preparation for suicide and subsequent support may compensate the risk posed by emotional closeness. Similarly, the impact of patient suicide is almost twice as high among the *concerned professionals* than among the *anticipators with support*, despite the fact that most of the professionals in both subgroups reported enough support.

When considering profiles, our results are both in line and discrepant with regard to previous findings.

The *anticipators with support* subgroup (A) is in line with the literature with regard to risk and protective factors. It has low risks such as less frequent confrontation with the suicidal scene, greater anticipation of the suicidal act and less frequent emotional closeness to the deceased patient, and high protection such as a greater percentages of people who had received sufficient support. Therefore, this group has the lowest stress reactions score.

The *distant professionals* (B), *no more contact with patient professionals* (C) and *concerned professionals* (D) subgroups have more challenging results with regard to risk and protective factors.

In the *distant professionals* subgroup (B), risk and protective factors are more contrasted and yet they lead to low stress reactions. The possible increase in stress reactions due to having seen the body of the deceased more frequently than in other subgroups or still being in contact with the patient at the time of death (risk factors) seems to have been balanced by the absence of emotional closeness, the high percentage of professionals having received sufficient support and the more frequent theoretical training received than in other subgroups (protective factors).

The *no more contact with patient professionals* subgroup (C) challenges findings of previous research. It has low risk factors (although suicide was not expected) such as no contact at the time of death, limited closeness to or responsibility for the patient, and protective factors such as sufficient support and more clinical training than colleagues in other subgroups. Nevertheless, professionals in this subgroup reported higher scores on IES-R than participants in subgroups A and B. Further research is needed to ascertain the influence of other possible variables (i.e., own suicidality, previous experience of completed suicide in the family, somatic or psychological issues previous to patient suicide, previous vulnerability, previous life event unrelated to patient suicide).

Findings on the largest subgroup, the *concerned professionals* (D) raise some questions. This subgroup, in comparison to the others ones, has high risk factors such as a close relationship to and responsibility for the patient, contact with him/her at time at death, a longer relationship to patient and working less frequently within an institution. It also has high protective factors such as having frequently sought and received sufficient support and being more experienced and older than colleagues in other subgroups [2, 10]. Nevertheless, these protective factors seem to have only partial effect on stress reduction. Further research is needed to ascertain whether other intervening variables, such as the nature and intensity of the support needed or other life event as well as personal factors, may be at play.

Consistent with the literature, the *unsupported professionals* subgroup (E) has the highest score on the IES-R. Professionals in this subgroup cumulate risk factors in all the groups of variables (relationship, exposure to suicide, support and training) and have no real protective factors. Professionals felt close to patient, were exposed to a higher number of suicides than their colleagues and received less theoretical and clinical training in suicide risk assessment than those in the other subgroups. Most particularly, all of them reported having received insufficient support although more than half of them had sought such support. This subgroup presents the highest percentage of nurses and women. In the literature, women have occasionally been found to be more impacted than men [14, 20, 21].

### Indications for education and practice

The combination of support and training seems to be crucial for mitigating risk factors. Professionals in the three low impacted subgroups had received the most theoretical or clinical training and sufficient support, while those in the most impacted subgroup had received the less training and support. Support or training alone seem to lessen stress reactions only partially, as shown by professionals in the *concerned professionals* subgroup (D), who received sufficient support but had low percentages of training. Therefore, theoretical education and clinical training combined with sufficient support should be provided to all professionals in a more systematic way [22], and should be accessible particularly to professionals who felt close to the deceased patient. Our findings reinforce previous research and recommendations about the necessity to provide training in suicide risk-assessment to mental health professionals in their educational curricula [11, 22].

More generally, the findings of this study suggest that for the low-impacted subgroups, postvention measures seem to have been appropriate with regard to the level of risk they were exposed to. Prevention and postvention measures seemed less adequate for the moderate impacted subgroup and insufficient for the high impacted subgroup. This raises further questions, because those professionals were the ones who sought out help most frequently. Institutional or vocational bodies' policies should encourage help-seeking strategies and take measures to ensure that professionals seeking help find it easily.

They should also make education and training available to all professionals, since recent studies [23–25] showed that education in postvention is likely to help professionals anticipate and cope with the consequences of a patient suicide and increase professionals’ confidence and feeling of competence with regard to emotional, clinical and medico-legal aspects following this event.

Taking into account the profiles of professionals and the severity of stress reactions may enable the screening of those caregivers most in need of support. This support should range from venting, exchanging with colleagues, superiors or supervisors for professionals in the low impacted subgroups, to specific counselling or therapeutic interventions for professionals with higher scores on the IES-R. Although the IES-R is not a diagnostic instrument, it could be useful as a self-screening tool for assessing symptomatic status and the first step to identify more vulnerable professionals, who could be then referred to colleagues for a throughout assessment of their condition (adjustment reaction, general distress, anxiety disorder, depression or PTSD). The well-being of professionals is indeed critical for their development and the pursuit of their career, as well as for the smooth running of the institution and most of all, for the detection, treatment and safety of suicidal patients. Postvention policies of organisational bodies or vocational associations after a patient suicide are crucial in this regard.

### Limitations and strength of the study

The findings ought to be interpreted in the light of their limitations and strengths. Firstly, this study relies on self-reported data. Results about stress reactions from the IES-R should therefore not be interpreted as clinical diagnosis. Variations in the IES-R score may reflect dispositional factors like personality traits, coping strategies, or peritraumatic emotionality or dissociation rather than situational factors, which may lead to interindividual differences in estimation regardless to patient suicide.

Secondly, the instrument itself could be too narrow to ascertain and discriminate the range of all the possible reactions following a patient suicide. Though the IES-R correlates with post-traumatic stress disorder [26], reactions observed in the present study may overlap with adjustment reactions, depression, anxiety disorder, or general distress.

Thirdly, there is a possible self-selection bias, since participation in the study was voluntary. As there are no available data on the population of professionals faced with a patient suicide who did not take part in the study, representativeness of the sample could not be examined. Professionals with more or less severe stress reactions might have chosen not to participate to the study for a number of reasons (not concerned or too concerned for example).

Fourthly, this study, as any previous one conducted so far, was retrospective in nature. Bias might arise in recalling stress reactions because the mean time elapsed since the most recent suicide was 4.2 years. However, similar or even greater time intervals are common in research studies about the impact of patient suicide among professionals [1, 27–29]. It should be mentioned that the average time interval was 3.1 years in the study of Weiss and Marmar [5] who designed and validated the IES-R.

Fifthly, this study focused on the most recent patient suicide in order to reduce recall bias as opposed to the most distressful suicide. This might have an influence on the intensity of the stress reactions since most of our respondents were faced with multiple completed suicides (desensitisation or cumulative effects).

Finally, the subgroups obtained may partly depend on the specific clustering computations that have been conducted. The results should nevertheless be suitable as the subgroups were clear-cut and stable as suggested by the validation tests.

This study has some strengths too. Firstly, to the best of our knowledge, it had the largest sample of professionals faced with a patient suicide, which allowed the systematic investigation of subgroups and their characteristics (clusters) for the first time.

Secondly, it investigated several vocations in two different work settings.

Thirdly, it used validated and well accepted tools of measurement (IES-R).

Fourthly, the sample was homogenous in terms of completed education: almost all professionals had earned the minimal required degree for practicing in their field. Previous studies sometimes found that professionals still in training felt greater impacts following a patient suicide [2, 10, 27].

## Conclusion

Three main contributions can be distinguished. Firstly, by identifying profiles, the study explored how the effects of predictors interacted, suggesting that their interplay rather than their isolate effects are decisive in differentiating professionals with regard to stress reactions (IES-R score). Indeed, stress reactions' scores varied according to the interplay of several variables pertaining to the relationship with the patient, exposure to suicide, support, training and some socio-demographic characteristics of respondents. The present study shows that risk and protective factors are not to be taken separately but to be considered within distinct profiles.

Secondly, cluster analysis made possible the identification of smaller subgroups' profiles (such as subgroup A the *anticipators with support* and subgroup E the *unsupported professionals*) which might get cushioned in a regression analysis.

Thirdly, the comprehensive view should contribute to improving the efficiency of prevention and postvention measures as well as training by taking into account risk profiles rather than considering the addition of isolated risk factors. Our findings suggest that professionals are able to cope with such an event if supported well enough or appropriately, i.e., according to their risk profile.
